# Hypothyroidism among Pregnant Women Attending the Outpatient Department of Obstetrics in a Tertiary Care Centre: A Descriptive Cross-sectional Study

**DOI:** 10.31729/jnma.8184

**Published:** 2023-06-30

**Authors:** Asmita Ghimire, Sailaja Ghimire, Prabha Baniya, Samriddha Raj Pant, Nilam Subedi, Poonam Koirala, Padam Raj Pant

**Affiliations:** 1 Department of Obstetrics and Gynecology, Tribhuvan University Teaching Hospital, Maharajgunj, Kathmandu, Nepal; 2 Department of Obstetrics and Gynecology, Bhim District Hospital, Bhairahawa, Nepal; 3 Department of Obstetrics and Gynecology, Grande International Hospital, Dhapasi, Kathmandu, Nepal

**Keywords:** *complications*, *hypothyroidism*, *miscarriage*, *pregnancy*, *thyroid*

## Abstract

**Introduction:**

Hypothyroidism is a common endocrine disorder occurring in pregnancy. Maternal and fetal complications are present in these patients. Timely identification and treatment help in the prevention of complications. The aim of this study was to find out the prevalence of hypothyroidism among pregnant women attending the Outpatient Department of Obstetrics in a tertiary care centre.

**Methods:**

A descriptive cross-sectional study was conducted in a tertiary care centre after taking ethical approval from the Institutional Review Committee (Reference number: 08/2021). Data from 1 January 2016 to 31 December 2020 were collected between 1 November 2021 to 31 November 2021. All pregnant women who had undergone thyroid level assessment in each trimester (first, second and third) and had delivered in the same centre were included in the study. However, pregnant women with comorbidities like hypertension, overt diabetes mellitus, hyperthyroidism, renal disease, cardiac disease, and neurological disorder were excluded. Convenience sampling method was used. Point estimate and 95% Confidence Interval were calculated.

**Results:**

Among 216 pregnant patients, the prevalence of hypothyroidism was 74 (34.25%) (27.9240.57, 95% Confidence Interval). Maternal complications were seen in 33 (44.59%). The commonest complication was oligohydramnios 10 (13.51%) followed by preterm delivery 8 (10.81%).

**Conclusions:**

The prevalence of hypothyroidism among pregnant women was found to be higher than other studies done in similar settings.

## INTRODUCTION

Hypothyroidism is the commonest endocrine disorder occurring in pregnancy with its profound and reversible effects on gland functions.^1,2^ In mothers various obstetric complications such as miscarriage, anaemia, hypertension, oligohydramnios, intrauterine growth retardation, preterm delivery, abruption and postpartum haemorrhage are seen. Whereas, in the fetus, it leads to stillbirth, perinatal death, neonatal intensive care unit admission and neonatal hypothyroidism.^3^

Studies done in different parts of Nepal among pregnant women show a high prevalence of thyroid disorders.^4,5^ Timely identification and treatment help in the prevention of complications.

The aim of this study was to find out the prevalence of hypothyroidism among pregnant women attending the Outpatient Department of Obstetrics in a tertiary care centre.

## METHODS

A descriptive cross-sectional study was conducted in the Outpatient Department of Obstetrics of Grande International Hospital, Dhapasi, Kathmandu, Nepal. Data from 1 January 2016 to 31 December 2020 were collected between 1 November 2021 to 31 November 2021. The ethical approval was obtained from the Institutional Review Committee of Grande International Hospital, Dhapasi, Kathmandu, Nepal (Reference number: 08/2021). All pregnant women who had undergone thyroid level assessment in each trimester (first, second and third) and had delivered in the Grande International Hospital were included in the study. However, pregnant women with comorbidities like hypertension, overt diabetes mellitus, hyperthyroidism, renal disease, cardiac disease, and neurological disorder were excluded. Convenience sampling method was used. The sample size was calculated using the following formula:


$$\begin{array}{ll} \text{n} & ={\text{Z}}^{2}\times \frac{\text{p}\times \text{q}}{{\text{e}}^{\text{2}}}\\ & =1.96^{2}\times \frac{0.50\times 0.50}{0.07^{2}}\\ & =196 \end{array}$$

Where,

n = minimum required sample size
Z = 1.96 at 95 % Confidence Interval (CI)
p = prevalence taken as 50% for maximum sample size calculation
q = 1-p
e = margin of error, 7%

The minimum required sample size was 196. However, the final sample size taken was 216. The reference ranges of the test values used in this study were as per the Guidelines of the American Thyroid Association for the diagnosis and management of thyroid disease during pregnancy. The normal value of thyroid level in first trimester is 0.10 to 2.50 mIU/l, second trimester is 0.20 to 3.00 mIU/l and third trimester is 0.30 to 3.00 mIU/l. Normal free T4 level is 0.70 to 1.80 ng/ml and free T3 level is 1.70 to 4.20 pg/ml.^6^

Data were entered and analysed using IBM SPSS Statistics version 22.0. Point estimate and 95% CI were calculated.

## RESULTS

Among 216 pregnant patients, the prevalence of hypothyroidism was 74 (34.25%) (27.92-40.57, 95% CI). The mean age of pregnant patients with hypothyroidism was 29.73±4.50. The mean TSH level of the first trimester was 3.98±4.96 mIU/l (Table 1).

**Table 1:** TSH level according to the trimester (n= 74).

Trimesters	Mean+SD (mIU/l)
First trimester	3.98±4.96
Second trimester	2.79±2.38
Third trimester	2.47±2.02

**Figure 1:**
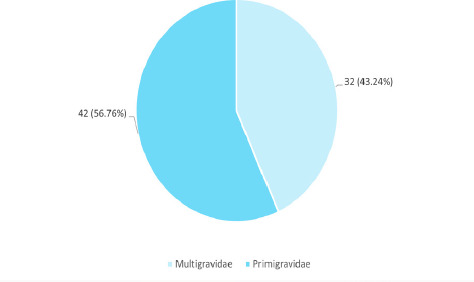
Parity among pregnant women with hypothyroidism (n= 74).

Among 216 pregnant patients, the history of miscarriage and treatment of fertility was seen in 26 (35.14%) and 19 (25.68%) respectively. Forty-two (56.76%) of the patients were primigravidae (Figure 1).

Oligohydramnios was the most common maternal complication 10 (13.51%) and 41 (55.41%) had no reported maternal complications (Table 2).

**Table 2:** Specific maternal complications (n = 74).

Complications	n (%)
Oligohydraminos	10 (13.51)
Preterm delivery	8 (10.81)
Hypertension	3 (4.05)
Hypertension + oligohydraminos	1 (1.35)
Obstetrics cholestasis	2 (2.70)
Cephalo pelvic disproportion	2 (2.70)
Premature rupture of membrane + oligohydraminos	2 (2.70)
Premature rupture of membrane + oligohydraminos + diabetes mellitus	1 (1.35)
Premature rupture of membrane + obstetric cholestasis	1 (1.35)
Placenta previa	1 (1.35)
Anaemia	1 (1.35)
Polyhydraminos	1 (1.35)
Diabetes mellitus	-
Premature rupture of membrane	-
None	41 (55.40)

## DISCUSSION

The prevalence of hypothyroidism in our study was found to be 74 (34.25%) which is higher than in similar studies. In a study done in a tertiary care centre in Kathmandu Nepal, the prevalence was found to be 329 (24.62%).^7^ A similar study done in another tertiary care centre had a lower prevalence than our study 5230 (25%).^8^ This disparity could be because the present study was conducted in a centre where there were many cases seeking fertility care and hypothyroidism is usually found to be higher in infertile groups of women.^9^

The mean age group was less than 30 years suggesting that even hypothyroidism occurs in the younger age group. This was similar to the study done in the population of Northern Spain.^10^ Higher distribution of history of spontaneous miscarriage rate 26 (35.13%) was seen in the present study. A study done in the population of Mumbai, India showed a miscarriage rate of 33 (9.29%). This could be because of the mechanism of fetal graft rejection.^11^ Contrary to the present study where 19 (25.67%) of patients received fertility treatment, the same study showed that only 11 (2.27%) patients received infertility treatment.^11^

Hypothyroidism causes increased prolactin leading to anovulation causing infertility.^9^ In the current study, the level was high as compared to another study. This could be because this study was conducted in a centre where a high number of infertility cases were investigated. The mean TSH level of women in the first trimester of hypothyroid was found to be more than 2.50 mIU/ml. Starting them on medication, controlled their thyroid status to a normal level as seen by evaluating these pregnant women in the second and third trimesters.

Oligohydramnios followed by preterm delivery was the most common complication. This was similar to the finding of a study done on Indian women where preterm delivery was a common complication.^12^ In a study done at the University of Texas Southwestern Medical Center, Dallas, Texas of stored serum samples from 17,298 women, maternal hypothyroidism was identified in 233 women (1.34%) and reports a higher rate of preterm delivery and preeclampsia which was different from our study.^13^ This disparity could be because all of the patients in our study had proper antenatal visits and preventable complications were well managed. In the high-risk group, prophylactic aspirin was started prior to 16 weeks period of gestation decreasing the risk of preeclampsia. However, in their study, they haven't mentioned the regularity of antenatal visits and preventable measures whether taken or not in the high-risk group.

There were a few limitations of the study. We did not have information on long-term outcomes because the study was retrospective. It is a single-centre study with a small sample size done in the Outpatient Department of Obstetrics in a short period of time.

## CONCLUSIONS

The prevalence of hypothyroidism among pregnant women was found to be higher than in other studies done in similar settings. Miscarriage rate, history of infertility treatment, and maternal and fetal complications were also found to be higher. The Nepalese government do not support universal screening of thyroid function among pregnant females. No guideline has been provided by the government to date.
